# Mechanisms of Exosome-Supplemented WJ-MSC Therapy in Degenerative Disc Disease: Integrating Proteomic Profiling with Clinical Functional Recovery

**DOI:** 10.3390/biomedicines14102235

**Published:** 2026-10-02

**Authors:** Alejandro Toro-Rivera, Wendy V. Jaraba-Álvarez, Angélica M. Calle-Ramírez, Juan P. Franco-Salazar, Luz Adriana Palacio, Daniela Martínez-Penaloza, Julián España, Karolynn Halpert, Hector Ortega-Arellano, Víctor Gómez

**Affiliations:** 1 BioXcellerator, Medellín 050021, Colombia; alejandro.toro@bioxcellerator.com (A.T.-R.); wendy.jaraba@bioxcellerator.com (W.V.J.-Á.); angelica.calle@bioxcellerator.com (A.M.C.-R.); adriana.palacio@bioxcellerator.com (L.A.P.); daniela.martinez@bioxcellerator.com (D.M.-P.); julianespana@yahoo.com (J.E.); karolynn@bioxcellerator.com (K.H.); 2 BioXtech, Medellín 050022, Colombia; juan.franco@bioxcellerator.com (J.P.F.-S.); hector.ortega@bioxcellerator.com (H.O.-A.)

**Keywords:** degenerative disc disease, WJ-MSCs, exosomes, proteomics, intradiscal therapy, Oswestry Disability Index, regenerative medicine

## Abstract

**Background**: Degenerative disc disease (DDD) is a primary cause of chronic back pain and disability globally. Although Wharton’s jelly-derived mesenchymal stromal cells (WJ-MSCs) possess proven therapeutic potential, the added clinical value of supplementing this treatment with their exosomal derivatives remains insufficiently defined in the literature. **Methods**: This study integrated the detailed proteomic characterization of these exosomes with clinical outcome analysis in a prospective cohort of 125 patients with thoracolumbar DDD treated intradiscally with WJ-MSCs alone or combined with exosomes. Exosomes were characterized using nanoparticle tracking analysis, flow cytometry, proteomics, and Gene Ontology enrichment analysis. **Results**: Proteomic profiling identified 1018 unique exosomal proteins significantly enriched in extracellular matrix organization, collagen and proteoglycan metabolism (highlighting structural effectors such as COL2A1 and lumican), proteolytic regulation (TIMP1, TIMP2, and CD109), and humoral immune and complement pathways (CD59 and SERPING1). Clinically, both treatment groups achieved significant reductions in pain and disability at 3 and 6 months compared to baseline. Supplementation with exosomes was not associated with clinically meaningful pain reduction at any follow-up (aRR = 0.836; 95% CI, 0.594–1.175; *p* = 0.3021). In contrast, WJ-MSCs plus exosomes were associated with a higher probability of clinically meaningful ODI improvement at any follow-up (aRR = 1.525; 95% CI, 1.075–2.162; *p* = 0.0180) and at 6 months (aRR = 2.003; 95% CI, 1.175–3.414; *p* = 0.0107). No serious adverse events were reported in either group. **Conclusions**: WJ-MSC-derived exosomes carry a multimodal protein cargo aligned with multiple biological processes involved in degenerative disc disease. Exosome supplementation was associated with greater functional improvement, but not pain reduction, supporting its potential role as a paracrine reinforcement strategy within WJ-MSC-based therapy and warranting further controlled translational studies.

## 1. Introduction

Mesenchymal stromal cells (MSCs) have become a central platform in regenerative medicine because of their capacity to shape the tissue microenvironment through immunomodulatory, anti-inflammatory, anti-apoptotic, trophic, and matrix-supportive mechanisms [1,2,3]. Although early therapeutic models emphasized cellular engraftment and differentiation, it is now increasingly recognized that many MSC-mediated effects are driven by paracrine signaling rather than durable tissue replacement. MSCs secrete cytokines, growth factors, extracellular matrix-associated proteins, regulatory lipids, and extracellular vesicles (EVs) that interact with resident cells and immune populations, thereby modulating inflammation, cell survival, angiogenesis, matrix turnover, and tissue repair [1,2,3,4]. Among EVs, exosomes have attracted particular interest because they carry bioactive proteins, lipids, messenger RNAs, and microRNAs capable of modifying recipient-cell behavior and amplifying specific components of MSC paracrine activity [5].

Human MSCs can be isolated from a variety of tissues, including bone marrow, adipose tissue, dental tissues, and perinatal sources such as umbilical cord and placenta. Although MSCs from these sources share common phenotypic characteristics and several functional properties, their tissue of origin can influence their proliferative capacity, immunomodulatory activity, secretory profile, and extracellular vesicle production and cargo [6,7,8]. Such source-dependent biological differences are particularly relevant when MSC-derived EVs are considered as therapeutic agents, because the cellular origin may contribute to differences in the composition and functional properties of the resulting vesicles. Among the available sources, umbilical cord-derived MSCs, particularly those isolated from Wharton’s jelly, are of translational interest because they can be obtained from perinatal tissue, exhibit high proliferative potential, and display relevant paracrine and immunomodulatory activity. These characteristics support the use of WJ-MSCs as the cellular source for the extracellular vesicles investigated in the present study [9,10,11].

This paracrine model is especially relevant in the context of intervertebral disc degeneration, where therapeutic success is unlikely to depend on a single regenerative mechanism. The degenerating disc represents a hostile and metabolically constrained microenvironment characterized by limited vascular supply, reduced nutrient diffusion, mechanical stress, inflammation, oxidative damage, and progressive extracellular matrix disruption [12,13]. Therefore, biological therapies for degenerative disc disease may require a multimodal mechanism of action capable of influencing resident nucleus pulposus and annulus fibrosus cells, inflammatory mediators, matrix-degrading enzymes, oxidative stress responses, and repair-associated signaling pathways (Figure 1).

Extracellular vesicles derived from MSCs may provide a biologically plausible strategy to enhance or complement cell-based therapy. In experimental models of intervertebral disc degeneration, MSC-derived exosomes have been reported to regulate nucleus pulposus cell apoptosis, inflammatory signaling, extracellular matrix metabolism, oxidative stress, mitochondrial function, and inflammasome-related pathways [14,15]. Mechanistically, these effects have been linked to signaling axes such as PTEN/PI3K/Akt, NLRP3 inflammasome regulation, mitochondrial homeostasis, and microRNA-mediated modulation of catabolic and inflammatory responses [14,15]. Thus, exosomes may function not only as acellular therapeutic candidates, but also as biologically active adjuncts capable of increasing the local concentration of selected paracrine signals when administered together with MSCs.

Degenerative disc disease is among the most common contributors to chronic low back pain and disability worldwide and has a major impact on quality of life, work productivity, and healthcare utilization [16,17]. Current clinical management, including pharmacological treatment, rehabilitation, image-guided interventions, and surgery, is primarily directed toward pain control, mechanical stabilization, or functional compensation. However, these approaches do not directly reverse the molecular and cellular processes that underlie disc degeneration. This limitation has driven increasing interest in intradiscal regenerative strategies designed to modify the biological niche of the degenerating disc rather than only addressing downstream symptoms.

The intervertebral disc is a complex fibrocartilaginous structure composed of the nucleus pulposus, annulus fibrosus, and cartilaginous endplates. Its mechanical performance depends on a specialized extracellular matrix enriched in proteoglycans, glycosaminoglycans, collagen, and water, which together preserve hydration, compressive resistance, tensile strength, and load distribution [12]. During degeneration, this homeostatic architecture is progressively disrupted. Loss of proteoglycans and hydration, altered collagen organization, reduced nucleus pulposus cell viability, mitochondrial dysfunction, oxidative stress, cellular senescence, and increased activity of matrix-degrading enzymes collectively contribute to impaired disc biomechanics and structural failure [12,13,18].

At the molecular level, degenerative disc disease is not merely a passive consequence of aging or mechanical wear, but an active biological process involving inflammatory, catabolic, and stress-response networks. Pro-inflammatory cytokines, including tumor necrosis factor-α, interleukin-1β, interleukin-6, and interleukin-17, promote extracellular matrix degradation, chemokine production, phenotypic changes in disc cells, apoptosis, pyroptosis, neurovascular ingrowth, and nociceptor sensitization [19]. These processes may establish a self-amplifying loop in which inflammation, oxidative stress, proteolytic imbalance, and matrix breakdown reinforce one another, contributing not only to structural degeneration but also to discogenic pain and disability [12,13,19].

Proteomic characterization of MSC-derived exosomes is therefore essential to understand whether their molecular cargo is aligned with disease-relevant mechanisms. This study aimed to characterize the protein cargo of WJ-MSC-derived exosomes and evaluate clinical outcomes in patients with thoracolumbar degenerative disc disease receiving intradiscal regenerative therapy. Patients treated with intradiscal WJ-MSCs alone were compared with those receiving WJ-MSCs combined with exosomes, with pain and functional disability assessed using the visual analog scale (VAS) and Oswestry Disability Index (ODI), respectively. By integrating exosomal proteomic characterization with patient-reported clinical outcomes, this study sought to explore the potential contribution of exosome supplementation to WJ-MSC-based intradiscal therapy and provide translational insights into its biological and clinical relevance.

## 2. Materials and Methods

### 2.1. Ethical Considerations

The study was approved by the Institutional Review Board of Clinisalud under approval number CEI-0324-07-2022 and was conducted in accordance with the bioethical principles established in the Declaration of Helsinki. All participants included in the study provided written informed consent before study participation.

### 2.2. Donor Screening and Cell Procurement

WJ-MSCs were isolated from umbilical cords obtained from healthy donors following informed consent and in accordance with established institutional procurement procedures, collected between April 2023 and December 2024. Donor eligibility was determined through clinical and epidemiological assessment and testing for transmissible infections. Serological and/or molecular testing was performed, as applicable, for hepatitis B virus, hepatitis C virus, human immunodeficiency virus, human T-lymphotropic virus, *Trypanosoma cruzi*, cytomegalovirus, *Toxoplasma gondii*, *Treponema pallidum*, and Epstein–Barr virus. Following donor qualification, the umbilical cord tissue was processed under controlled conditions, and the cells were isolated from Wharton’s jelly according to the institutional manufacturing protocol.

### 2.3. Cell Culture, Expansion, and Cryopreservation

To isolate Wharton’s jelly-derived mesenchymal stromal cells (WJ-MSCs), the umbilical cord is removed from the transport solution and washed with Dulbecco’s Phosphate-Buffered Saline (DPBS) (Biowest, Nuaillé, France, Ref. L0930-500) to eliminate residual blood. The umbilical arteries and vein are carefully removed, and the remaining Wharton’s jelly tissue is dissected into approximately 2–3 mm fragments. Tissue explants are placed in Petri dishes and allowed to adhere to the culture surface. Opti-MEM (Gibco, Grand Island, NY, USA, Ref. 31985070) supplemented with 20% heat-inactivated fetal bovine serum (FBS) (Biowest, Nuaillé, France, Ref. S140B) is then added. Cultures are maintained at 37 °C in a humidified atmosphere containing 5% CO_2_ for approximately 15 days under standard culture conditions. The cultures are monitored regularly for changes in medium appearance, including color shifts and turbidity.

Once cell migration from the explants is observed, adherent cells are harvested using TrypLE™ Select (Gibco, Grand Island, NY, USA, Ref. 12563-029). The isolated cells are subsequently expanded in T300 culture flasks and BioFactory™ 5- and 10-layer cell culture systems (NEST, Wuxi, China, Cat. No. 771204 and 771302 respectively) using low-glucose DMEM (Biowest, Nuaillé, France, Ref. L0066) supplemented with 10% human platelet lysate (hPL) (PTLMax Sigma Aldrich, Burlington, MA, USA, Ref. SCM141). Cell cultures are expanded until passages 6–7 and are cryopreserved at a concentration of 2.5 × 10^6^ cells/mL in a cryopreservation medium consisting of FBS supplemented with 10% dimethyl sulfoxide (DMSO) (WAK-Chemie Medical GmbH, Steinbach, Germany). Cryopreserved cell stocks are stored in the vapor phase of liquid nitrogen until further use.

All MSC and exosome batches underwent rigorous sterility controls, including testing for endotoxins, mycoplasma and microbial contamination, to ensure suitability for downstream applications.

### 2.4. WJ-MSC Marker Expression

WJ-MSCs were characterized according to the minimum criteria proposed by the International Society for Cellular Therapy [20]. WJ-MSC identity and phenotype were characterized by flow cytometry. Mesenchymal markers (CD90, CD73, and CD105, BD Biosciences, San Diego, CA, USA, Cat. Nos. 312204, 353004, and 349504, respectively) and non-mesenchymal markers (CD45, CD34, CD11B, CD19, and HLA-DR, BD Biosciences San Diego, CA, USA, Cat. Nos. 555595, 550257, and 560839, respectively) were assessed using an Epics XL Flow Cytometer (Beckman Coulter, Beckman Coulter, Brea, CA, USA), with data analyzed in FlowJo v7.6.2. Positive marker expression was defined as fluorescence exceeding the corresponding negative-control threshold in >99% of the cell population. IgG1 PE-Cy5.5, IgG1-PE, and IgG1-FITC isotype controls (BD Biosciences, San Diego, CA, USA) were used as negative controls.

### 2.5. Differentiation of WJ-MSCs

#### 2.5.1. Adipogenic Differentiation

For adipogenic differentiation, passage 2–3 cells are seeded at a density of 2000 cells/cm^2^ in 24-well plates using standard culture medium. After 72 h, the culture medium is replaced with adipogenic differentiation medium consisting of αMEM supplemented with an adipogenic differentiation supplement (R&D Systems^®^, Minneapolis, MN, USA, Catalog #CCM007). The medium is changed every 3–4 days. Control cells are maintained in standard culture medium. Following 21 days of induction, cells are fixed with 4% paraformaldehyde (PFA) and incubated with an anti-mFABP4 antibody (Human Mesenchymal Stem Cell Functional Identification Kit, R&D Systems, Minneapolis, MN, USA, Catalog Number SC006). Adipogenic differentiation is subsequently evaluated by microscopic assessment of adiponectin protein expression.

#### 2.5.2. Osteogenic Differentiation

For osteogenic differentiation, passage 2–3 cells are seeded at a density of 4000 cells/cm^2^ in 24-well plates using standard culture medium. Upon reaching 90–100% confluence, the culture medium is replaced with osteogenic induction medium consisting of αMEM supplemented with an osteogenic differentiation supplement (R&D Systems^®^, Minneapolis, MN, USA, Catalog #CCM007). The medium is refreshed every 3–4 days. After 21 days of induction, cells are fixed with 4% PFA and incubated with an anti-human osteocalcin antibody (Human Mesenchymal Stem Cell Functional Identification Kit, R&D Systems, Minneapolis, MN, USA, Catalog Number SC006). Osteogenic differentiation is then confirmed by microscopic evaluation of osteocalcin protein expression.

#### 2.5.3. Chondrogenic Differentiation

For chondrogenic differentiation, passage 2–3 cells are seeded at a density of 2000 cells/cm^2^ in 24-well plates using standard culture medium. Once 90–100% confluence is achieved, the culture medium is replaced with chondrogenic induction medium consisting of D-MEM/F-12 basal medium (Gibco) supplemented with a chondrogenic differentiation supplement (R&D Systems^®^, Minneapolis, MN, USA, Catalog #CCM007). The medium is changed every 2–3 days. Control cells are maintained in standard culture medium. After 21 days of induction, cells are fixed with 4% PFA and incubated with an anti-human aggrecan antibody (Human Mesenchymal Stem Cell Functional Identification Kit, R&D Systems, Minneapolis, MN, USA, Catalog Number SC006). Chondrogenic differentiation is subsequently assessed by microscopic evaluation of aggrecan protein expression.

### 2.6. Exosome Isolation and Characterization

Exosome isolation and characterization were performed using the previously established workflow [21], with the procedure summarized below. WJ-MSC cultures at 75–85% confluence underwent a 24-h hPL deprivation period to promote extracellular vesicle release while minimizing hPL-derived contaminants. Cultures were washed twice with DPBS and maintained in serum-free, low-glucose DMEM for 24 h at 37 °C and 5% CO_2_; hypoxic cultures were maintained at 5% O_2_. Conditioned media were collected aseptically and stored at −80 °C until processing.

For exosome purification, conditioned media were centrifuged at 800× *g* for 30 min and processed by single-pass tangential flow filtration (TFF; Pellicon^®^ Single-Pass Tangential Flow Filtration system, Merck Millipore, Darmstadt, Germany) using sterile polyethersulfone hollow-fiber membranes with a 10-kDa molecular weight cut-off (Repligen, Spectrum Labs, Waltham, MA, USA, Cat. No. C02-E500-05-N). Membranes were pre-rinsed with three volumes of sterile PBS (pH 7.4), and samples were processed at an inlet flow rate of 100 mL/min, maintaining feed-stream shear force below 2000 s^−1^. The material was concentrated 30-fold and diafiltered 10 times with saline. Subsequently, 10 mL of the TFF product was purified by size-exclusion chromatography using a pre-equilibrated qEV Original 70 nm+ column (Izon Science Limited, Christchurch, New Zealand) containing 10 mL of 20 mM HEPES (pH 7.4) (Sigma-Aldrich, St. Louis, MO, USA, Cat. No. H3375). Purified exosomes were recovered in saline and stored at −80 °C.

Particle size distribution and concentration were assessed by nanoparticle tracking analysis (NTA) using a NanoSight NS300 (Malvern Panalytical, Malvern, Worcestershire, UK), following dilution of samples (1:100–1:1000) in sterile saline. Measurements were performed using 500 µL of sample, 60-s acquisition periods, and three capture replicates. Flow cytometry was used to assess particle size and the presence of CD9, CD63, and CD81 (BD Biosciences, San Diego, CA, USA, Cat. Nos. 312204, 353004, and 349504, respectively) using a DxFLEX cytometer (Beckman Coulter, Brea, CA, USA).

For proteomic characterization, exosomes were lysed in 50 mM Tris (pH 7.5) (Sigma-Aldrich, Merck, Darmstadt, Germany, Cat. No. 11853973001), 100 mM NaCl (Sigma-Aldrich, Merck, Darmstadt, Germany, Cat. No. S3014), and 4% SDS (Bio-Rad Laboratories, Hercules, CA, USA, Cat. No. 1610301) containing protease and phosphatase inhibitors (Sigma-Aldrich, Merck, Darmstadt, Germany, Cat. No. 4693159001), followed by incubation at 95 °C for 10 min and lyophilization. Cysteines were reduced with TCEP (Thermo Scientific, Pierce, Waltham, MA, USA, Cat. No. 20490) and alkylated with MMTS (Thermo Scientific, Pierce, Waltham, MA, USA, Cat. No. 23011), and proteins were digested with trypsin ((Biowest, Nuaillé, France), Cat. No. L0940-500) using S-Trap columns (Protifi, Fairport, NY, USA). Peptides were analyzed by nano-HPLC–MS/MS on an Exploris 240 Orbitrap (Thermo Fisher Scientific, Waltham, MA, USA) using a C18 column (Thermo Scientific, Waltham, MA, USA, Cat. No. 87784) (75 µm × 50 cm) with a 90-min gradient. The 20 most intense precursor ions within the 375–1200 *m*/*z* range were selected for MS/MS fragmentation.

### 2.7. Clinical Outcome Measures

To evaluate clinical outcomes, an observational, longitudinal, prospective cohort study was conducted among patients diagnosed with degenerative disc disease who were treated intradiscally with WJ-MSCs and their derivatives at a regenerative medicine clinic in Medellín, Colombia. The patient inclusion period extended from October 2024 to August 2025. A census-based study design was employed, including all eligible patients treated during the predefined study period who met the prespecified inclusion criteria and did not meet any exclusion criteria. Medical records and follow-up assessments (clinical scales) were reviewed for the included patients. All participants provided informed consent before the use and analysis of their clinical information. Data was anonymized to ensure confidentiality, and the study complied with institutional protocols and international standards for research ethics and data protection. This study was conducted in accordance with the Strengthening the Reporting of Observational Studies in Epidemiology (STROBE) guidelines.

Patients were classified into two treatment groups according to the intervention received. The first group included patients treated with WJ-MSCs alone, whereas the second group included patients treated with WJ-MSCs in combination with exosomes. Treatment selection was based on individual clinical assessment by the treating neurosurgeons and the institutional treatment protocols in effect at the time of the procedure.

### 2.8. Eligibility Criteria

Patients were eligible for inclusion if they were 18 years of age or older and had been considered candidates for WJ-MSC therapy; eligible records corresponded to patients with a clinical diagnosis of discogenic pain and imaging-confirmed degenerative disc disease.

Patients were included if they had dorsolumbar pain lasting more than 3 months associated with imaging-confirmed degenerative disc disease involving the T1–S1 spinal levels. Eligible patients were required to have at least one intervertebral disc with degenerative changes corresponding to Pfirrmann grade II to IV on magnetic resonance imaging, and the affected disc had to be included among the intradiscally treated levels. In addition, patients were required to have clinical symptoms consistent with discogenic pain and to have completed baseline functional assessment scales before treatment, with an Oswestry Disability Index (ODI) score of ≥20 points and/or a Visual Analog Scale (VAS) score of ≥4 for thoracic or lumbar disc disease.

Patients were excluded if they had not provided informed consent for the use of their clinical data in observational research at the time of treatment. Records were also excluded when data from either of the two baseline scales were unavailable, when more than two follow-up scale measurements were missing, or when missing data exceeded 20% for the variables of interest. Additional exclusion criteria included inconsistent or incoherent clinical information, insufficient information to determine the intervention received or evaluate the outcomes of interest, and non-legitimate outliers in quantitative variables when correction was not possible after review of the medical record.

### 2.9. Intervention

Patients received intradiscal WJ-MSC therapy according to the anatomical spinal region treated. Patients in the WJ-MSC-only group received intradiscal WJ-MSCs without exosome supplementation, whereas patients in the combined-treatment group received intradiscal WJ-MSCs together with umbilical cord-derived exosomes.

For lumbar discs, the protocol consisted of a single intradiscal dose of 20 × 10^6^ WJ-MSCs per disc. In patients receiving combined therapy, 2 mL of exosomes containing 3 × 10^9^ particles were additionally administered intradiscally as a single dose.

For thoracic discs, the protocol consisted of a single intradiscal dose of 10 × 10^6^ WJ-MSCs per disc. In patients receiving combined therapy, 2 mL of exosomes containing 3 × 10^9^ particles were additionally administered intradiscally as a single dose.

The cellular dose, exosome dose, administration volume, and procedural conditions were standardized according to the institutional protocols in effect at the clinical center. All procedures were performed by trained physicians under controlled clinical conditions and with imaging guidance.

### 2.10. Outcome Measures

The primary clinical outcomes were pain intensity and functional disability. Pain intensity was measured using the Visual Analog Scale (VAS), ranging from 0 to 10, where 0 represents no pain and 10 represents the worst pain imaginable. Functional disability was evaluated using the Oswestry Disability Index (ODI), a patient-reported questionnaire designed to assess disability related to low back pain across daily activities, including pain intensity, personal care, lifting, walking, sitting, standing, sleeping, social life, traveling, and employment or homemaking activities. ODI scores range from 0 to 100, with higher scores indicating greater disability. Clinical outcomes were assessed at baseline and during follow-up evaluations performed at 3 and 6 months after treatment. Safety was evaluated using a questionnaire to document adverse events at 24–72 h and at two weeks, three months, and six months following treatment.

### 2.11. Definition of Clinically Meaningful Improvement

Clinically meaningful pain improvement was defined as a reduction of at least 4 points in VAS from baseline. Clinically meaningful functional improvement was defined as a reduction of at least 10 points in ODI from baseline.

A global responder variable was defined to identify patients who achieved the predefined threshold for clinically meaningful improvement at any available follow-up assessment (3 or 6 months). Additionally, binary responder variables were defined separately for the 3- and 6-month follow-up assessments.

### 2.12. Statistical Analysis

#### 2.12.1. Proteomic Data Processing and Functional Analysis

Proteomic data were processed using Proteome Discoverer v2.5 (Thermo Fisher Scientific, Waltham, MA, USA) and Mascot Server v2.8 (Matrix Science Ltd., London, UK).by matching MS/MS spectra against the human UniProt-SwissProt database with a decoy search strategy. Peptide identifications were accepted at an FDR < 1% (high confidence), with MMTS cysteine alkylation as a fixed modification and N-terminal acetylation, methionine oxidation, and Gln-to-pyro-Glu conversion as variable modifications. To assess exosomal enrichment, identified proteins were compared with a curated panel of 34 high-confidence exosomal markers from ExoCarta and EVpedia, with detection of ≥9 markers (≥25% of the reference panel) considered supportive of exosomal enrichment.

Functional enrichment analysis was subsequently performed using the ClusterProfShinyGSEA web-based platform (accessed 30 September 2024), which implements the *clusterProfiler* framework in R (version 4.12.0). Gene Ontology (GO) enrichment was evaluated across the Biological Process (BP), Cellular Component (CC), and Molecular Function (MF) domains using *Homo sapiens* annotations and Entrez Gene identifiers. Multiple testing was controlled using the Benjamini–Hochberg method and GO terms with an adjusted *p*-value < 0.05 were considered significantly enriched.

#### 2.12.2. Clinical Data Analysis

A descriptive analysis of the study population was performed. Continuous variables were summarized as means and standard deviations or as medians with interquartile ranges, according to their distribution, verified through the Shapiro–Wilk test. Categorical variables were presented as absolute and relative frequencies.

Because the outcome variables did not meet the normality assumption, longitudinal within-group comparisons between baseline and follow-up assessments were performed using the Wilcoxon signed-rank test. Effect sizes for these within-group comparisons were estimated using the *r* statistic.

Clinically meaningful improvement was additionally analyzed as a binary responder outcome. Because thresholds for clinically important change vary according to the clinical population, baseline symptom severity, outcome instrument, scale range, and method used to estimate the minimal important change, responder thresholds were selected based on published evidence in low back pain and spinal populations. For disability, an ODI responder was defined as a patient achieving a reduction of ≥10 points from baseline. This threshold is consistent with the 10-point absolute minimal important change proposed by international consensus for the ODI and falls within the range of clinically important changes reported in low back pain and lumbar spine populations [22,23,24]. For pain intensity, a VAS responder was defined as a patient achieving a reduction of ≥4 points from baseline. This more conservative threshold was selected to identify a substantial absolute improvement in pain rather than the minimum perceptible change, considering that published estimates of clinically important change in chronic low back pain vary according to the analytical method and have reached approximately 4 points on 0–10 pain scales [24,25]. Responder outcomes were evaluated at any available follow-up and separately at 3 and 6 months. Responder outcomes were evaluated at any available follow-up and separately at 3 and 6 months of assessment. Associations between treatment groups and responder status were assessed using Poisson regression with robust variance, and crude and adjusted risk ratios (RRs) with 95% confidence intervals (CIs) were estimated.

Crude models included treatment group as the only independent variable, while adjusted models included treatment group, age, and sex. This approach was selected because the outcomes were binary, and event frequencies were greater than 10%, making odds ratios less appropriate due to the potential overestimation of the association’s magnitude. Analyses were conducted using the available paired observations for each outcome and follow-up assessment. All statistical tests were two-sided, and a *p*-value below 0.05 was considered statistically significant. All statistical analyses were performed using RStudio for Windows (Posit Software, PBC; version 2025.09.2, Build 418; “Cucumberleaf Sunflower” release).

## 3. Results

### 3.1. WJ-MSCs and Exosome Characterization and Sterility

The cells demonstrated plastic adherence under standard culture conditions and expressed CD73, CD90, and CD105 in at least 95% of the analyzed cell population. In contrast, the expression of CD14, CD19, CD34, CD45, and HLA-DR was limited to no more than 2% of the analyzed cell population. The multipotent differentiation capacity of the cells was confirmed through their ability to differentiate into osteogenic, adipogenic, and chondrogenic lineages using specificin vitro differentiation assays. Exosome preparations showed appropriate size, concentration, and expression of the characteristic surface markers CD9, CD63, and CD81 (Supplementary Figure S1).

Sterility, endotoxin, mycoplasma, and microbial contamination controls were also satisfactory for all evaluated batches.

### 3.2. Functional Characterization of the Exosomal Protein Cargo

Proteomic analysis identified 1018 unique proteins in the exosomal preparation, based on the combined protein lists contributing to the Biological Process, Cellular Component, and Molecular Function Gene Ontology analyses. The identified protein cargo was subsequently subjected to Gene Ontology enrichment analysis to characterize its predominant biological functions and molecular activities.

Gene Ontology (GO) enrichment analysis was performed across Biological Process, Cellular Component, and Molecular Function categories. The full GO analysis is provided in Supplementary Table S1. Overall, enriched terms clustered predominantly into functional domains related to extracellular matrix organization and remodeling, collagen and proteoglycan metabolism, regulation of proteolytic activity, inflammatory and complement-associated pathways, cellular stress responses, angiogenesis, and TGF-β-associated signaling.

The most prominent functional domain was related to extracellular matrix organization and homeostasis. GO analysis showed strong enrichment of terms associated with extracellular matrix organization, collagen fibril organization, collagen metabolism, collagen biosynthesis, and extracellular matrix structural components. Consistent enrichment was also observed for collagen-containing extracellular matrix and collagen trimer within the Cellular Component category. Together, these findings indicate that extracellular matrix- associated functions constitute a major component of the biological profile represented within the exosomal cargo.

A second major domain involved proteoglycan and glycosaminoglycan metabolism. Enriched terms included glycosaminoglycan metabolic and biosynthetic processes, as well as proteoglycan metabolic processes. These findings further support the representation of extracellular matrix composition and matrix homeostasis within the exosomal functional profile, which is relevant to the maintenance of the hydrated matrix environment of the nucleus pulposus.

The analysis also identified a prominent domain related to extracellular matrix turnover and proteolytic regulation. Significant enrichment was observed for extracellular matrix disassembly and for multiple terms related to regulation and inhibition of peptidase activity. Rather than indicating a unidirectional increase in matrix degradation, this pattern suggests that the exosomal cargo contains a broad repertoire of functions involved in the regulation of extracellular proteolysis and matrix remodeling.

Another relevant domain comprised immune and inflammatory regulation, particularly complement-associated processes. Strong enrichment was observed for complement activation, the classical complement pathway, and regulation of complement activation, together with terms related to acute inflammatory responses and regulation of inflammation. This profile indicates that immune and inflammatory regulatory functions are substantially represented within the exosomal cargo.

GO enrichment additionally identified a cellular stress-response domain, characterized predominantly by processes related to oxidative stress, reactive oxygen species responses, and reactive oxygen species metabolism. This functional profile suggests that modulation of cellular redox homeostasis represents another biological property associated with the exosomal cargo.

Finally, angiogenesis and TGF-β-associated signaling represented additional enriched functional domains. Enrichment of terms related to regulation of angiogenesis included both positive and negative regulatory processes, suggesting a context-dependent regulatory profile rather than a unidirectional pro-angiogenic effect. TGF-β-related terms, including cellular responses to TGF-β and TGF-β receptor signaling, were also significantly enriched, indicating representation of this pathway within the exosomal protein cargo. Given the pleiotropic and context-dependent functions of TGF-β, this enrichment may reflect potential involvement in tissue remodeling, extracellular matrix regulation, and cellular responses rather than a specific pro- or anti-inflammatory effect. Overall, the GO enrichment profile revealed a multifunctional exosomal cargo predominantly associated with extracellular matrix homeostasis and remodeling, inflammatory regulation, cellular stress responses, and tissue regenerative signaling. The distribution of representative proteins across these interconnected functional domains is summarized in Figure 2.

### 3.3. Pathophysiology-Oriented Molecular Function Enrichment Analysis

To further explore the relevance of the exosomal cargo in the context of degenerative disc disease, a pathophysiology-oriented GO molecular function analysis was performed. This analysis showed strong enrichment of extracellular matrix and cell matrix interaction-related functions (Supplementary Table S2). Among the most significant terms, cadherin binding was the top enriched molecular function, involving 93 proteins (FDR = 2.64 × 10^−39^), followed by extracellular matrix structural constituent activity, which included 64 proteins (FDR = 1.14 × 10^−35^).

Additional matrix-associated functions included collagen binding, extracellular matrix binding, structural molecule activity conferring elasticity, and extracellular matrix structural constituent activity conferring tensile strength and compression resistance. These enriched functions included several proteins directly related to disc matrix architecture and biomechanical integrity, such as COL1A1, COL1A2, COL2A1, COL3A1, COL4A1, COL4A2, COL5A1, COL5A2, COL6A1, COL6A2, COL6A3, COL12A1, DCN, BGN, LUM, HAPLN1, VCAN, HSPG2, FN1, SPARC, THBS1, THBS2, TNC, POSTN, FBN1, and FBN2.

In addition, glycosaminoglycan binding was highly enriched, involving 56 proteins (FDR = 3.79 × 10^−20^), together with proteoglycan binding, heparin binding, heparan sulfate proteoglycan binding, hyaluronic acid binding, and chondroitin sulfate binding. These findings support a functional profile associated with extracellular matrix organization, proteoglycan and glycosaminoglycan interactions, and maintenance of the hydrated matrix environment that is central to nucleus pulposus homeostasis. This pathophysiology-oriented distribution of enriched Molecular Function terms is shown in Figure 3.

The analysis also identified enriched molecular functions related to matrix remodeling, growth factor signaling, immune regulation, oxidative stress response, and mechanotransduction. Matrix remodeling and protease regulation were represented by peptidase regulator activity, endopeptidase inhibitor activity, peptidase inhibitor activity, enzyme inhibitor activity, endopeptidase activity, protease binding, serine-type endopeptidase activity, and metallopeptidase activity, involving proteins such as TIMP1, TIMP2, SERPINE1, SERPINE2, SERPING1, A2M, MMP2, ADAM9, ADAM10, ADAM17, ADAMTS1, HTRA1, CTSB, and CTSL. Growth factor-related functions were also enriched, including growth factor binding, platelet-derived growth factor binding, insulin-like growth factor binding, transforming growth factor beta binding, and cytokine binding, with relevant proteins such as IGFBP3, IGFBP4, IGFBP5, IGFBP6, LTBP1, LTBP2, LTBP3, TGFBI, ENG, THBS1, and CD109.

Immune-related enrichment included antigen binding, immunoglobulin receptor binding, complement binding, opsonin binding, and MHC protein complex binding, whereas antioxidant activity and oxidoreductase-related functions reflected a potential response to oxidative stress. Overall, these findings suggest that the exosomal cargo contains a biologically coherent molecular signature aligned with key mechanisms of degenerative disc disease, including extracellular matrix disruption, proteoglycan loss, abnormal matrix remodeling, inflammatory regulation, oxidative stress, altered cell adhesion, and mechanotransduction (Figure 3).

### 3.4. Key Molecular Functions Related to Degenerative Disc Disease

To further highlight the most biologically relevant Molecular Function terms, a key term lollipop plot was generated. This visualization emphasized the main enriched functions related to degenerative disc disease pathophysiology, particularly those associated with extracellular matrix structure, proteoglycan/GAG homeostasis, matrix remodeling/protease regulation, cell adhesion/mechanotransduction, immune/complement regulation, oxidative stress response, and growth factor/cytokine signaling.

The key functions represented in this analysis included glycosaminoglycan binding, extracellular matrix structural constituent activity, collagen binding, extracellular matrix binding, endopeptidase inhibitor activity, peptidase regulator activity, metallopeptidase activity, integrin binding, growth factor binding, antioxidant activity, complement binding, hyaluronic acid binding, transforming growth factor beta binding, and cytokine binding. Together, these selected terms reinforce the presence of a functional exosomal signature associated with extracellular matrix organization, proteoglycan and glycosaminoglycan interactions, regulation of proteolytic remodeling, oxidative stress modulation, immune/complement regulation, and growth factor-mediated tissue remodeling. These key Molecular Function terms are summarized in Figure 4.

### 3.5. Clinical Results

A total of 125 patients were included in the study, with varying availability of follow-up data across assessment time points. The overall mean age was 50.33 ± 12.24 years, with a median age of 49 years (range, 22–83). The cohort was predominantly male, comprising 95 patients (76.0%), whereas 30 patients (24.0%) were female. Regarding treatment allocation, 69 patients (55.2%) received WJ-MSC therapy alone, while 56 patients (44.8%) received WJ-MSCs combined with exosomes (Table 1).

When analyzed according to intervention group, patients treated with WJ-MSCs alone had a mean age of 48.52 ± 10.87 years and a median age of 49 years (range, 25–72). Patients who received WJ-MSCs combined with exosomes were older on average, with a mean age of 52.55 ± 13.51 years and a median age of 50.5 years (range, 22–83). Both groups were predominantly male; however, the proportion of male patients was higher in the WJ-MSC-only group (81.16%) than in the WJ-MSC plus exosomes group (69.64%). Conversely, the proportion of female patients was higher in the combined-therapy group (30.4%) than in the WJ-MSC-only group (18.8%) (Table 1).

Among the 125 patients included in the study, lumbar intradiscal treatment was performed in 119 patients (95.2%), including 66 of 69 patients (95.7%) in the WJ-MSC-alone group and 53 of 56 (94.6%) in the WJ-MSC plus exosome group. Thoracic treatment was performed in 14 patients (11.2%), with seven patients in each treatment group. Treatment across thoracic and lumbar regions was not mutually exclusive (Table 2).

The distribution of treated intervertebral levels reflected lower lumbar involvement. Among patients receiving lumbar treatment, L4–L5 and L5–S1 were the most frequently treated levels, each treated in 104 of 119 patients (87.4%), followed by L3–L4 (83/119, 69.7%), L2–L3 (67/119, 56.3%), and L1–L2 (51/119, 42.9%). Thoracic treatment was distributed across multiple levels, with T6–T7 being the most frequently treated level (7/14, 50.0%). T3–T4, T4–T5, T7–T8, and T10–T11 were each treated in 6 of 14 patients (42.9%). Because patients could undergo treatment at multiple intervertebral levels, level-specific frequencies were not mutually exclusive (Table 3).

Baseline MRI findings demonstrated predominantly advanced degenerative changes in the lumbar spine. Modic type II changes were the most frequently documented lumbar Modic abnormality, occurring in 66 of 119 patients (55.5%), whereas type I and type III changes were documented in 9 (7.6%) and 1 (0.8%) patients, respectively. Forty patients (33.6%) had no documented lumbar Modic changes. Pfirrmann grade IV was the most frequently documented grade (75 patients, 63.0%), followed by grade V (33, 27.7%) and grade III (11, 9.2%) (Table 4).

Among the 14 patients who underwent thoracic treatment, Modic type II changes were documented in 4 (28.6%), while 9 (64.3%) had no Modic changes. Thoracic degeneration was characterized predominantly by Pfirrmann grades III and IV, documented in 5 (35.7%) and 9 (64.3%) patients, respectively. No thoracic patients had documented Pfirrmann grade II or V findings (Table 4).

Disability, as assessed using the Oswestry Disability Index (ODI), decreased during follow-up in both treatment groups. In the WJ-MSC-only group, the median ODI decreased from 17.50 (Q1–Q3, 14.50–21.50) at baseline to 10.00 (3.00–12.00) at 3 months and 8.00 (4.00–12.00) at 6 months. Similarly, in the WJ-MSC + exosomes group, the median ODI decreased from 17.50 (14.00–26.00) at baseline to 8.00 (3.00–13.00) at 3 months and 7.00 (3.00–10.00) at 6 months. Compared with baseline, ODI scores were significantly lower at both 3 and 6 months in the WJ-MSC-only group (both *p* < 0.001) and in the WJ-MSC + exosomes group (both *p* < 0.001) (Table 5).

Pain intensity, as assessed using the visual analog scale (VAS), also decreased during follow-up in both treatment groups. Among patients treated with WJ-MSCs alone, the median VAS score decreased from 8.00 (Q1–Q3, 7.00–9.00) at baseline to 4.00 (1.00–5.00) at 3 months and 5.00 (2.00–7.00) at 6 months. In the WJ-MSC + exosomes group, the median VAS score decreased from 8.00 (6.00–9.00) at baseline to 3.00 (2.00–5.00) at 3 months and 4.00 (2.00–5.00) at 6 months. Compared with baseline, VAS scores were significantly lower at both 3 and 6 months in the WJ-MSC-only group (both *p* < 0.001) and in the WJ-MSC + exosomes group (both *p* < 0.001) (Table 5).

When changes from baseline were compared between groups, reductions in ODI scores were observed with both treatments, with a greater magnitude of change in the WJ-MSC plus exosomes group. At 3 months, the median change in ODI was −7 points in the WJ-MSC-only group compared with −12 points in the WJ-MSC plus exosomes group, corresponding to a between-group difference in median change of −5 points. At 6 months, the median change was −8 points in the WJ-MSC-only group and −12 points in the combined-therapy group, corresponding to a difference of −4 points. However, these between-group differences did not reach statistical significance at either 3 months (*p* = 0.1018; *r* = 0.1889) or 6 months (*p* = 0.0887; *r* = 0.2596), although a trend toward greater improvement in disability was observed in the WJ-MSC plus exosomes group (Table 6).

With respect to VAS-measured pain intensity, both groups showed similar reductions from baseline. At 3 months, the median change was −4 points in both groups (difference in medians, 0), whereas at 6 months, the median change was −4 points in the WJ-MSC-only group and −3 points in the WJ-MSC plus exosomes group, corresponding to a difference of 1 point. Statistically significant between-group differences were not observed at either follow-up time point (3 months: *p* = 0.8959; *r* = 0.0146; 6 months: *p* = 0.5863; *r* = 0.0821), suggesting a comparable magnitude of change between interventions in terms of pain intensity reduction (Table 6).

For clinically meaningful improvement in pain intensity, defined as a ≥4-point reduction in VAS, no statistically significant differences were observed between treatment groups. In the any-follow-up analysis, VAS response was observed in 57.14% of patients in the WJ-MSC plus exosomes group and 68.18% of patients in the WJ-MSC-only group. Exosome supplementation was not associated with a greater probability of clinically meaningful pain reduction in either the crude model (RR = 0.838; 95% CI, 0.602–1.167; *p* = 0.2951) or the age- and sex-adjusted model (aRR = 0.836; 95% CI, 0.594–1.175; *p* = 0.3021). Consistent with this finding, no statistically significant between-group differences were observed at the 3- or 6-month follow-up assessments (Table 7).

In contrast, clinically meaningful functional improvement, defined as a ≥10-point reduction in ODI, was more frequently observed in the WJ-MSC plus exosomes group. In the any-follow-up analysis, combined therapy was associated with a significantly higher probability of ODI response in both crude (RR = 1.524; 95% CI, 1.071–2.169; *p* = 0.0194) and adjusted models (aRR = 1.525; 95% CI, 1.075–2.162; *p* = 0.0180). At 3 months, the crude analysis showed a significant association favoring the combined-treatment group (65.85% vs. 41.18%; RR = 1.599; 95% CI, 1.011–2.529; *p* = 0.0446), although this association did not remain statistically significant after adjustment for age and sex (aRR = 1.462; 95% CI, 0.924–2.313; *p* = 0.1048). But, when at 6 months analysis was made, patients receiving WJ-MSCs plus exosomes had approximately twice the probability of achieving clinically meaningful ODI improvement compared with those receiving WJ-MSCs alone, both in the crude model (RR = 1.979; 95% CI, 1.149–3.407; *p* = 0.0138) and in the adjusted model (aRR = 2.003; 95% CI, 1.175–3.414; *p* = 0.0107) (Table 7).

Regarding treatment safety, all participants (*n* = 125) completed at least one adverse event questionnaire, and no serious adverse events (SAEs) were reported. The proportion of participants experiencing non-serious adverse events (AEs) was comparable between the treatment groups. Overall, 82.61% (*n* = 57) of participants who received WJ-MSC alone (*n* = 69) and 80.36% (*n* = 45) of those who received the combined WJ-MSC plus exosome therapy (*n* = 56) reported one or more adverse events, with no statistically significant difference between the groups (*p* = 0.747). A total of 228 AEs were reported, of which 133 (58.33%) occurred in the WJ-MSC-only group and 95 (41.67%) in the WJ-MSC plus exosome group. Local symptoms predominated in both groups, accounting for 209 AEs in total. Most events occurred within the first 24–72 h (*n* = 190), with 113 events (59.47%) reported in the WJ-MSC group and 77 (40.53%) in the WJ-MSC plus exosome group. Pain at the injection site and back pain were the most frequently reported symptoms in both groups (Table 8).

At the 15-day follow-up, 28 adverse events were reported across both groups, with an equal distribution between the WJ-MSC-only group and the WJ-MSC plus exosome group (14 events (50%) each). Local symptoms accounted for 21 (75%) of these events. Pain at the injection site and back pain remained the most frequently reported symptoms during this period. Between 3 and 6 months of follow-up, a considerable decrease in the frequency of adverse events was observed, with a total of 10 AEs reported: 6 (60%) in the WJ-MSC group and 4 (40%) in the WJ-MSC plus exosome group. During this period, new-onset and persistent back pain were the most frequently reported adverse events in both groups. All adverse events resolved without the need for major interventions (Table 8).

## 4. Discussion

This translational study integrated proteomic characterization of WJ-MSC-derived exosomes with clinical outcome analysis in patients with thoracolumbar degenerative disc disease treated with intradiscal regenerative therapy. The main findings were: (i) the exosomal protein cargo showed functional enrichment in biological processes relevant to disc degeneration. (ii) Both treatment groups experienced improvements in pain and functional disability during follow-up. (iii) Exosome supplementation was not associated with a greater probability of clinically meaningful pain improvement but was associated with a higher probability of clinically meaningful functional improvement, particularly at 6 months and across available follow-up assessments. Together, these findings suggest that the exosome supplementation may contribute more clearly to functional recovery than to additional analgesia when combined with WJ-MSCs therapy.

The biological plausibility of these findings is supported by our previous in vitro study, in which hypoxia-conditioned WJ-MSC-derived exosomes promoted fibroblast proliferation, increased COL1A2 expression, and reduced β-galactosidase activity in senescent fibroblasts. These findings, together with the present proteomic analysis, support the concept that WJ-MSC-derived exosomes may influence several processes relevant to disc homeostasis rather than acting through a single molecular pathway. Degenerative disc disease involves interconnected alterations in extracellular matrix integrity, cellular senescence, oxidative stress, inflammation, apoptosis, and tissue remodeling [10,11,13,14]. Accordingly, the multimodal functional profile identified here may be particularly relevant to the complex metabolically constrained environment of the degenerating disc.

Extracellular matrix organization and collagen-associated functions represented an important component of the proteomic profile. The intervertebral disc depends on a specialized extracellular matrix to maintain hydration, compressive resistance, tensile strength, and load distribution [13,26]. During degeneration, loss of proteoglycans and water content, disruption of collagen organization, and progressive remodeling of the matrix contribute to impaired mechanical function [26,27]. The enrichment of extracellular matrix structural constituents, collagen-associated proteins, and matrix-binding proteins in the exosomal cargo is therefore consistent with a potential role in matrix organization and tissue remodeling. The presence of proteins involved in collagen fibrillogenesis, matrix organization, and cell–matrix interactions further supports the biological relevance of this signature. Importantly, these findings should be interpreted as evidence of molecular cargo with potential relevance to matrix homeostasis rather than as direct evidence that exosomes restore disc structure in vivo.

The enrichment of protease-regulatory functions provides an additional mechanism by which the exosomal cargo could interact with the degenerative microenvironment. Disc degeneration is characterized by increased extracellular matrix turnover and excessive catabolic activity involving matrix metalloproteinases and ADAMTS enzymes, contributing to degradation of collagen, aggrecan, and other matrix components [27]. The identification of peptidase regulators, endogenous protease inhibitors, and matrix-associated regulatory proteins is therefore compatible with a potential role in modulating excessive proteolysis. This should not, however, be interpreted as complete inhibition of matrix-degrading enzymes, because physiological matrix turnover is necessary for tissue remodeling. Rather, the proteomic profile suggests the possibility of a regulatory influence on the balance between matrix degradation and preservation. Such modulation could be relevant to functional recovery because matrix stability influences mechanical tolerance, tissue integrity, and mobility [26,28].

The immune-related signature provides another layer of interpretation. The enrichment of humoral and complement-associated processes, together with the presence of complement components and regulatory proteins, suggests that the exosomal cargo may interact with inflammatory pathways within the degenerating disc. Complement activation has been implicated in cellular senescence, tissue damage, and Modic changes associated with disc degeneration [9,10,29,30]. The coexistence of complement components with regulatory proteins such as CD59 and SERPING1 is compatible with a role in maintaining immune balance rather than simply promoting or suppressing complement activity. This interpretation is also consistent with the broader literature describing immunomodulatory, anti-inflammatory, and tissue-protective effects of MSC-derived extracellular vesicles in degenerative disc disease [31,32,33,34]. However, because no post-treatment molecular measurements were available in the present study, these proteomic findings cannot establish that these pathways were functionally modulated in vivo.

Oxidative stress and cellular stress-response pathways may further contribute to the biological relevance of the exosomal cargo. The degenerating disc is characterized by hypoxia, impaired nutrient diffusion, acidic conditions, mitochondrial dysfunction, and increased reactive oxygen species, all of which may contribute to cellular senescence, apoptosis, and impaired matrix maintenance [26,32,35,36,37]. The enrichment of processes related to oxidative stress and reactive oxygen species responses suggests that the exosomal cargo contains proteins potentially relevant to cellular adaptation within this hostile microenvironment. Similarly, enrichment of TGF-β-associated signaling may represent a cross-cutting mechanism connecting matrix synthesis, remodeling, inflammatory regulation, and cell survival [38,39]. Experimental studies have shown that TGF-β-related signaling can have anabolic and anti-catabolic effects in degenerative disc models [40,41,42]. Nevertheless, TGF-β activity is context-dependent, and excessive or dysregulated signaling may also contribute to fibrosis or maladaptive remodeling [38,43,44]. Therefore, the presence of TGF-β-associated proteins should be interpreted as part of a broader remodeling signal rather than as evidence of a purely regenerative effect. Likewise, the angiogenesis-related component of the proteomic profile may be relevant because the healthy disc is largely avascular and aneural in its inner regions, whereas degeneration is associated with abnormal vascular and neural ingrowth [45,46,47,48,49,50]. The present findings may therefore reflect a capacity to interact with pathways involved in vascular and tissue remodeling, although their functional consequences cannot be determined from proteomic data alone.

The clinical findings provide an important translational context for these molecular observations. Both treatment groups showed significant within-group improvements in VAS and ODI, consistent with the growing clinical literature supporting the potential of intradiscal MSC-based therapies for degenerative disc disease and chronic discogenic low back pain [51]. However, exosome supplementation was not associated with a greater probability of clinically meaningful pain improvement, whereas the combined WJ-MSC plus exosomes group showed a higher probability of clinically meaningful functional improvement, particularly at 6 months and across available follow-up assessments after adjustment for age and sex. This distinction is relevant because pain intensity and functional disability represent related but distinct dimensions of degenerative disc disease. Pain may be influenced by inflammatory activity, mechanical loading, nociceptor sensitization, analgesic use, rehabilitation, physical activity, and psychosocial factors, whereas ODI captures the broader consequences of low back pain on daily activities and physical functioning [9,10,48,52,53].

The absence of a superior VAS response should therefore not be interpreted as evidence of absent biological activity. WJ-MSCs may already exert substantial paracrine effects on inflammatory and pain-related pathways, potentially limiting the measurable additional analgesic effect of exosome supplementation. In contrast, functional recovery may reflect more integrated and longer-term changes involving matrix homeostasis, mechanical tolerance, mobility, and tissue remodeling. From this perspective, the combined therapy may represent a form of paracrine reinforcement, in which viable WJ-MSCs provide dynamic and environmentally responsive cellular signaling while exosomes provide a concentrated extracellular vesicle-associated molecular cargo [1,3]. The observed association between combined therapy and functional improvement is compatible with this model, although the observational design does not permit attribution of causality or demonstration of synergy between the two components.

The safety findings provide additional clinical context. No serious adverse events were reported, and the frequency of non-serious adverse events was comparable between treatment groups. Most adverse events consisted of local symptoms occurring during the first 24–72 h, particularly injection-site and back pain, and resolved without major intervention. These findings are consistent with previous clinical evidence suggesting generally acceptable short-term tolerability of intradiscal MSC-based approaches, although the available literature is heterogeneous. A randomized study comparing allogeneic mesenchymal precursor cells with placebo reported low rates of treatment-emergent and serious adverse events, whereas a systematic review and meta-analysis by Xie et al. [54] reported no significant differences in several adverse-event outcomes between MSC therapy and comparator treatments but identified higher odds of treatment-related adverse events in one analysis (OR = 3.05, 95% CI: 1.11–8.40; *p* = 0.03). Differences in cell products, treatment protocols, patient populations, and comparator interventions should be considered when comparing these findings. Accordingly, the present results support short-term tolerability in this cohort but do not establish long-term safety.

This study has several strengths. It integrates molecular characterization of WJ-MSC-derived exosomes with patient-centered clinical outcomes and directly compares WJ-MSC therapy with WJ-MSC therapy combined with exosomes. The use of VAS and ODI, together with predefined responder thresholds and crude and age- and sex-adjusted relative risks, provides clinically interpretable estimates of treatment-associated outcomes. Importantly, the integration of proteomic and clinical data allows a translational interpretation of the findings while maintaining a distinction between molecular plausibility and demonstrated biological effects.

Crucially, the future success of these translational pathways hinges on biomanufacturing scalability. Traditional 2D static culture platforms suffer from limited yield, batch-to-batch variation, and phenotypic drift. To transition toward definitive Phase II/III clinical trials, advanced manufacturing approaches must be adopted. Dynamic 3D bioprocessing—such as hollow-fiber bioreactors and automated stirred-tank systems—enables high-density cell expansion under controlled physiological conditions (hypoxia, shear stress regulation), vastly increasing EV secretion kinetics and yield per donor batch. Furthermore, downstream processing must integrate automated, closed-system continuous Tangential Flow Filtration (TFF) coupled with size-exclusion chromatography (SEC) to preserve EV structural integrity and multimodal protein cargo while efficiently removing culture contaminants. Implementing these field-defining manufacturing technologies will be essential to ensure lot-to-lot consistency, clinical-grade purity, and commercial viability for future interventional trials in spinal regenerative medicine.

Several limitations must also be acknowledged. The prospective observational design and non-randomized treatment allocation preclude causal inference and leave the possibility of residual confounding. Relevant clinical and radiological variables, including symptom duration, number and anatomical distribution of treated discs, baseline Pfirrmann grade, Modic changes, previous spinal surgery, analgesic use, rehabilitation, occupational demands, and comorbidities, were not consistently available or incorporated into the adjusted models. In addition, variation in follow-up availability and attrition at 6 months may have introduced selection bias and reduced the precision of the clinical estimates. These limitations should be considered when interpreting the association between exosome supplementation and functional improvement.

The proteomic analysis also characterized the administered exosomal product rather than the biological response of the treated disc. Thus, enrichment of specific pathways does not demonstrate their activation or modulation in vivo. No post-treatment molecular biomarkers, imaging biomarkers, or tissue analyses were available to directly connect the exosomal cargo with biological changes following intradiscal administration. Similarly, structural outcomes such as disc hydration, disc height, Modic changes, or quantitative MRI parameters were not systematically evaluated longitudinally. Therefore, the proposed mechanistic relationships remain in biologically plausible interpretations rather than demonstrated causal mechanisms.

Future studies should address these limitations through randomized designs with standardized treatment allocation and longer follow-up. Imaging-based endpoints, including Pfirrmann grade progression, disc height, T2 mapping, endplate integrity, quantitative disc hydration, and Modic changes, would help determine whether functional improvement is accompanied by structural or compositional changes. Biomarker studies assessing inflammatory cytokines, matrix degradation products, oxidative stress markers, and extracellular vesicle-associated signatures may further clarify whether the proteomic pathways identified in the exosomal cargo are reflected in patient-level biological responses. Future analyses should also consider dose–response relationships, the number and anatomical distribution of treated discs, baseline disease severity, and responder phenotypes. Given the distinct molecular and functional profiles observed in this study, a potential randomized clinical trial could compare four groups: (i) WJ-MSCs alone, representing the cellular therapy component; (ii) WJ-MSC-derived exosomes alone, representing an acellular extracellular vesicle-based therapy; (iii) combined WJ-MSCs plus exosomes, as evaluated in the present study; and (iv) an active control receiving an established interventional therapy for pain management, with the specific intervention determined according to local clinical practice.

## 5. Conclusions

This study highlights the translational potential of a two-component regenerative strategy combining WJ-MSCs as dynamic, responsive paracrine cellular mediators with their exosome derivatives as a concentrated extracellular vesicle-associated molecular cargo. The proteomic landscape of WJ-MSC-derived exosomes revealed a multimodal functional profile encompassing extracellular matrix organization, collagen and proteoglycan metabolism, regulation of proteolytic activity, inflammatory and complement pathways, oxidative stress responses, angiogenic regulation, and TGF-β-associated tissue remodeling. These findings indicate that the exosomal cargo is functionally aligned with multiple interconnected processes underlying degenerative disc disease. Clinically, WJ-MSC therapy was associated with improvements in pain and disability, while the addition of exosomes was associated with a higher probability of clinically meaningful functional improvement, particularly at six months, but not with superior pain reduction. Together, these findings support the potential of exosome supplementation as a paracrine reinforcement strategy within WJ-MSC-based therapy and provide a translational framework linking exosomal molecular cargo with patient-centered outcomes. Prospective controlled studies integrating molecular, imaging, and clinical endpoints are warranted to further validate these mechanisms and determine the therapeutic contribution of exosome supplementation.

## Figures and Tables

**Figure 1:**
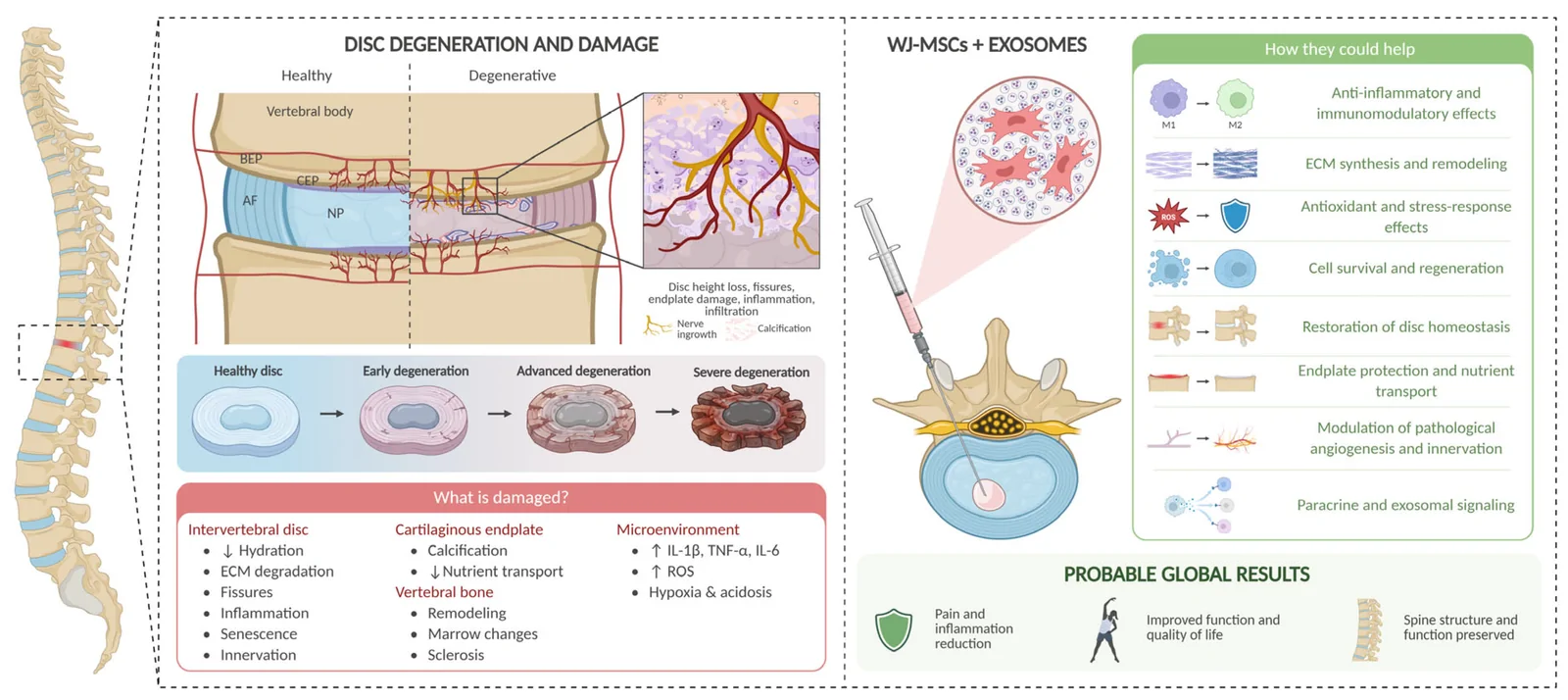
Pathophysiology of intervertebral disc degeneration and potential therapeutic mechanisms of WJ-MSCs and exosomes. Schematic overview of the main degenerative changes in the intervertebral disc, the proposed mechanisms of WJ-MSCs and their exosomes, and the expected outcomes. AF, annulus fibrosus; CEP, cartilaginous endplate; ECM, extracellular matrix; NP, nucleus pulposus; ROS, reactive oxygen species. “↑” and “↓” indicate overexpression and underexpression, respectively. Created in BioRender. SAS, B. (2026) https://BioRender.com/lr4t07x.

**Figure 2:**
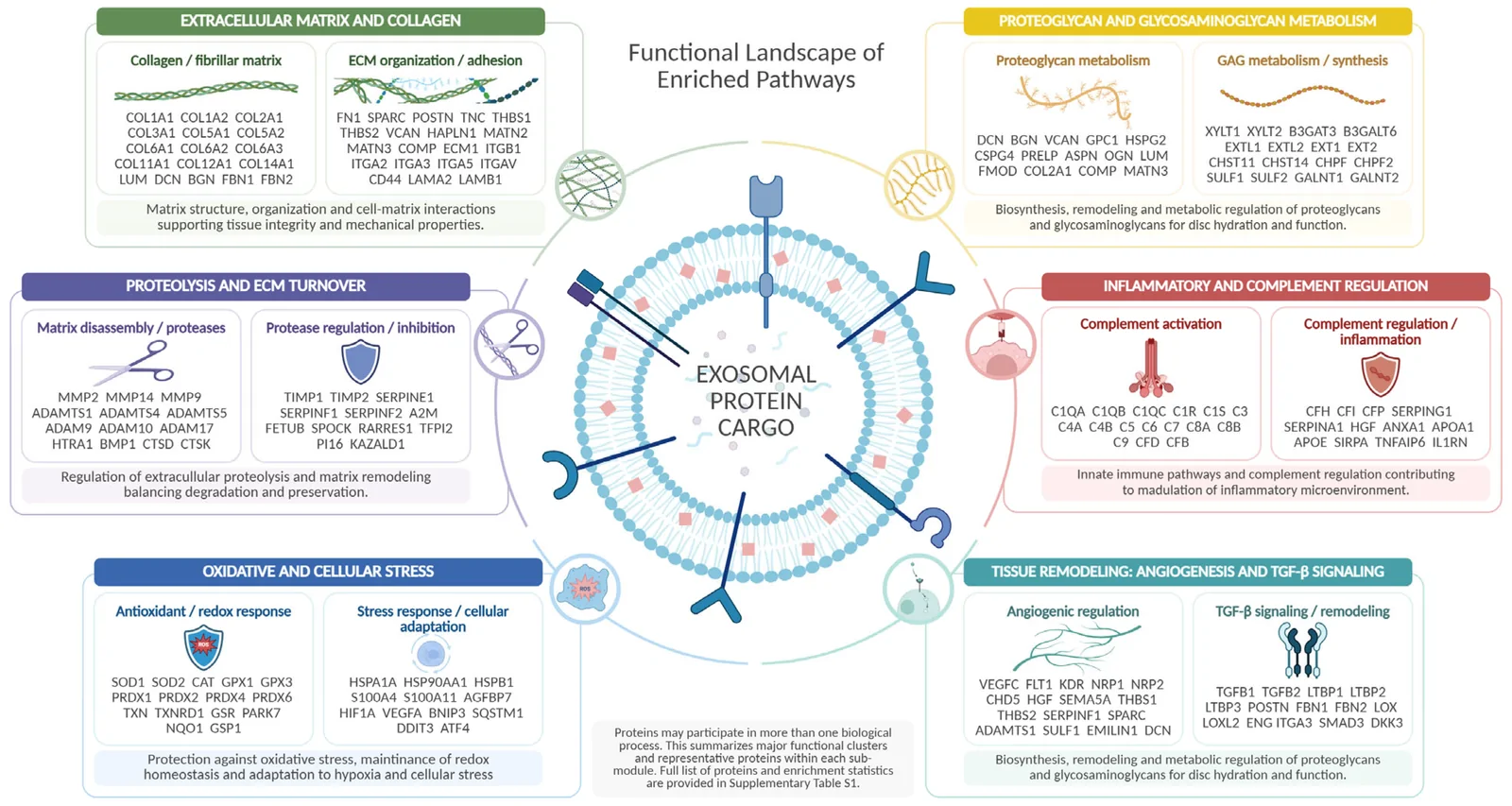
Functional landscape of enriched pathways represented by the exosomal protein cargo. Summary of the main biological processes enriched among the proteins identified in WJ-MSC-derived exosomes in the present study. The results are grouped into six functional categories related to extracellular matrix organization, proteoglycan and glycosaminoglycan metabolism, proteolysis, inflammatory regulation, cellular stress responses, and tissue remodeling. Proteins may contribute to more than one biological process. Detailed protein lists and pathway annotations are provided in Supplementary Table S1. Created in BioRender. SAS, B. (2026) https://BioRender.com/lr4t07x.

**Figure 3:**
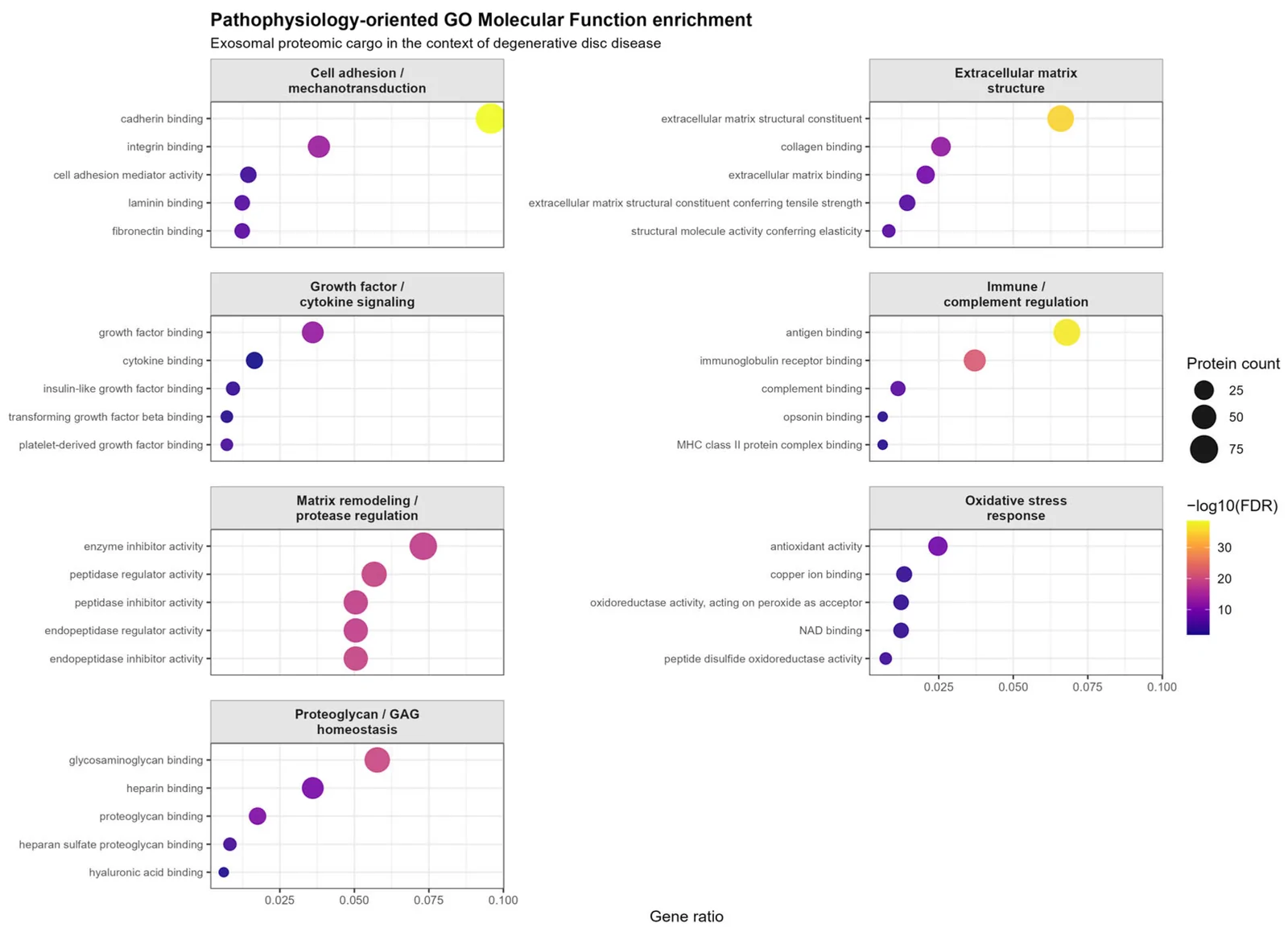
Pathophysiology-oriented GO molecular function enrichment of the exosomal protein cargo. Dot plot showing the main enriched Gene Ontology (GO) Molecular Function terms selected according to their relevance to degenerative disc disease. Enriched functions are grouped into biological themes including cell adhesion and mechanotransduction, extracellular matrix structure, growth factor and cytokine signaling, immune and complement regulation, matrix remodeling and protease regulation, oxidative stress response, and proteoglycan/GAG homeostasis. Dot size represents the number of proteins associated with each term, while color indicates statistical significance based on −log10(FDR). Gene ratio represents the proportion of identified exosomal proteins associated with each molecular function.

**Figure 4:**
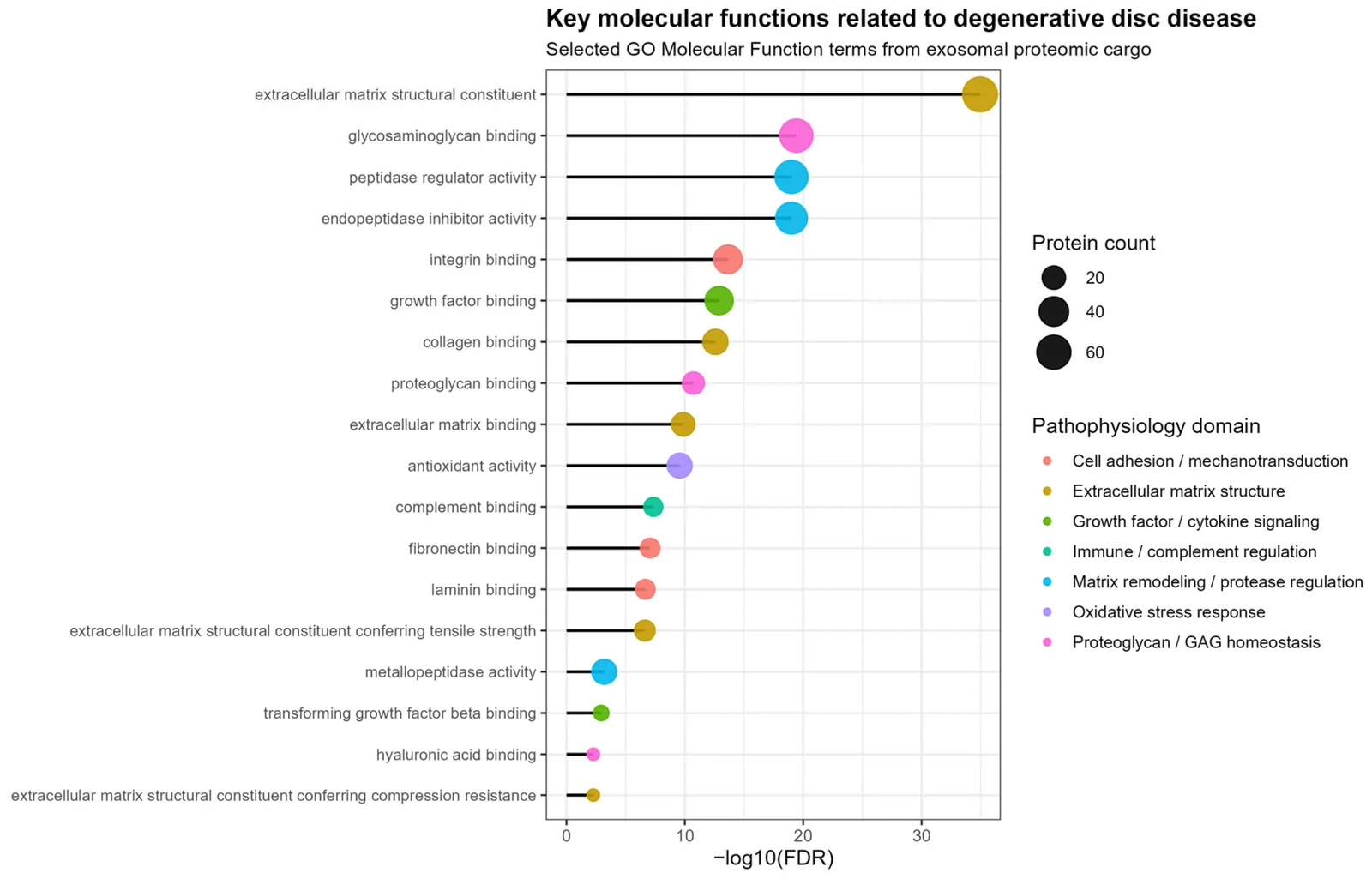
**Key molecular functions associated with degenerative disc disease.** Lollipop plot highlighting selected GO Molecular Function terms from the exosomal proteomic cargo according to their relevance to degenerative disc disease pathophysiology. Terms are grouped into functional domains encompassing extracellular matrix structure, proteoglycan and glycosaminoglycan homeostasis, matrix remodeling and protease regulation, cell adhesion and mechanotransduction, growth factor and cytokine signaling, immune and complement regulation, and oxidative stress response. The x-axis represents statistical significance as −log10(FDR), while bubble size indicates the number of proteins associated with each molecular function. Together, these functions highlight the potential involvement of exosomal cargo in matrix organization, proteolytic regulation, cellular interactions, inflammatory modulation, oxidative stress responses, and tissue remodeling.

**Table 1:** Baseline demographic characteristics of the overall cohort and according to treatment group.

Characteristic	Overall Cohort	WJ-MSC Only	WJ-MSC + Exosomes	*p*-Value
Unique patients, *n* (%)	125	69 (55.2%)	56 (44.8%)	
Age, mean ± SD	50.33 ± 12.24	48.52 ± 10.87	52.55 ± 13.51	0.0735 ^†^
Age, median	49	49	50.5	—
Age, range	22–83	25–72	22–83	—
Male sex, *n* (%)	95 (76.0%)	56 (81.16%)	39 (69.64%)	0.1338 ^††^
Female sex, *n* (%)	30 (24.0%)	13 (18.84%)	17 (30.36%)	0.1338 ^††^

^†^ Age was compared between groups using Welch’s *t*-test. ^††^ Sex distribution was compared using the chi-square test.

**Table 2:** Anatomical Distribution of Thoracic and Lumbar Intradiscal Treatment.

Anatomical Region	Overall Cohort (*N* = 125)	WJ-MSCs Alone (*n* = 69)	WJ-MSCs + Exosomes (*n* = 56)
Thoracic	14 (11.2%)	7 (10.1%)	7 (12.5%)
Lumbar	119 (95.2%)	66 (95.7%)	53 (94.6%)

Table note: Values are presented as *n* (%). Patients could receive treatment in more than one anatomical region; therefore, categories are not mutually exclusive. WJ-MSCs, Wharton’s jelly-derived mesenchymal stromal cells.

**Table 3:** Distribution of Treated Thoracic and Lumbar Intervertebral Levels by Treatment Group.

Intervertebral Level	Overall	WJ-MSCs Alone	WJ-MSCs + Exosomes
Thoracic	*N* = 14	*n* = 7	*n* = 7
T1–T2	1 (7.1%)	1 (14.3%)	0 (0.0%)
T2–T3	5 (35.7%)	3 (42.9%)	2 (28.6%)
T3–T4	6 (42.9%)	4 (57.1%)	2 (28.6%)
T4–T5	6 (42.9%)	4 (57.1%)	2 (28.6%)
T5–T6	5 (35.7%)	4 (57.1%)	1 (14.3%)
T6–T7	7 (50.0%)	5 (71.4%)	2 (28.6%)
T7–T8	6 (42.9%)	4 (57.1%)	2 (28.6%)
T8–T9	4 (28.6%)	1 (14.3%)	3 (42.9%)
T9–T10	5 (35.7%)	1 (14.3%)	4 (57.1%)
T10–T11	6 (42.9%)	1 (14.3%)	5 (71.4%)
T11–T12	2 (14.3%)	0 (0.0%)	2 (28.6%)
T12–L1	2 (14.3%)	0 (0.0%)	2 (28.6%)
Lumbar	*N* = 119	*n* = 66	*n* = 53
L1–L2	51 (42.9%)	29 (43.9%)	22 (41.5%)
L2–L3	67 (56.3%)	34 (51.5%)	33 (62.3%)
L3–L4	83 (69.7%)	46 (69.7%)	37 (69.8%)
L4–L5	104 (87.4%)	57 (86.4%)	47 (88.7%)
L5–S1	104 (87.4%)	55 (83.3%)	49 (92.5%)

Table note: Values are presented as *n* (%), with percentages calculated using the number of patients treated in the corresponding anatomical region. Patients could receive treatment at more than one intervertebral level; therefore, levels are not mutually exclusive, and percentages may sum to more than 100%. Each patient was counted only once per intervertebral level. WJ-MSCs, Wharton’s jelly-derived mesenchymal stromal cells.

**Table 4:** Baseline Thoracic and Lumbar MRI Characteristics: Modic Changes and Pfirrmann Grades.

MRI Characteristic	Overall	WJ-MSCs Alone	WJ-MSCs + Exosomes
Thoracic spine	*N* = 14	*n* = 7	*n* = 7
Modic changes			
Type I	0 (0.0%)	0 (0.0%)	0 (0.0%)
Type II	4 (28.6%)	1 (14.3%)	3 (42.9%)
Type III	0 (0.0%)	0 (0.0%)	0 (0.0%)
Unspecified	1 (7.1%)	0 (0.0%)	1 (14.3%)
None	9 (64.3%)	6 (85.7%)	3 (42.9%)
Pfirrmann grade			
Grade II	0 (0.0%)	0 (0.0%)	0 (0.0%)
Grade III	5 (35.7%)	4 (57.1%)	1 (14.3%)
Grade IV	9 (64.3%)	3 (42.9%)	6 (85.7%)
Grade V	0 (0.0%)	0 (0.0%)	0 (0.0%)
Lumbar spine	*N* = 119	*n* = 66	*n* = 53
Modic changes			
Type I	9 (7.6%)	5 (7.6%)	4 (7.5%)
Type II	66 (55.5%)	39 (59.1%)	27 (50.9%)
Type III	1 (0.8%)	0 (0.0%)	1 (1.9%)
Unspecified	3 (2.5%)	3 (4.5%)	0 (0.0%)
None	40 (33.6%)	19 (28.8%)	21 (39.6%)
Pfirrmann grade			
Grade II	1 (0.8%)	0 (0.0%)	1 (1.9%)
Grade III	11 (9.2%)	7 (10.6%)	4 (7.5%)
Grade IV	75 (63.0%)	43 (65.2%)	32 (60.4%)
Grade V	33 (27.7%)	16 (24.2%)	17 (32.1%)

Table note: Values are presented as *n* (%), with percentages calculated using the number of patients treated in the corresponding anatomical region. MRI, magnetic resonance imaging; WJ-MSCs, Wharton’s jelly-derived mesenchymal stromal cells.

**Table 5:** Pain intensity and functional disability outcomes over time according to the treatment group.

Outcome	Treatment Group	Baseline		3 Months			6 Months		
		*n*	Median (Q1–Q3)	*n*	Median (Q1–Q3)	*p*-Value ^1^	*n*	Median (Q1–Q3)	*p*-Value ^1^
ODI	WJ-MSCs only	68	17.50 (14.50–21.50)	35	10.00 (3.00–12.00)	<0.001	22	8.00 (4.00–12.00)	<0.001
	WJ-MSCs + exosomes	56	17.50 (14.00–26.00)	41	8.00 (3.00–13.00)	<0.001	21	7.00 (3.00–10.00)	<0.001
VAS	WJ-MSCs only	69	8.00 (7.00–9.00)	38	4.00 (1.00–5.00)	<0.001	23	5.00 (2.00–7.00)	<0.001
	WJ-MSCs + exosomes	56	8.00 (6.00–9.00)	42	3.00 (2.00–5.00)	<0.001	21	4.00 (2.00–5.00)	<0.001

Table note: Values are presented as median (Q1–Q3). The number of available observations is reported separately for each outcome, treatment group, and follow-up time point. The decrease in sample size over time reflects the use of available follow-up data at each assessment, as not all patients had completed or available outcome measurements at 3 or 6 months. Therefore, the reported *n* varies across outcomes and time points. ^1^ *p*-values correspond to within-group comparisons between each follow-up assessment and baseline and were calculated using the Wilcoxon signed-rank test for paired samples. ODI, Oswestry Disability Index; VAS, visual analog scale; WJ-MSCs, Wharton’s jelly-derived mesenchymal stromal cells.

**Table 6:** Between-group comparison of paired changes from baseline in pain intensity and functional disability.

Outcome	Follow-Up	WJ-MSC Only, Paired *n*	WJ-MSC + Exosomes, Paired *n*	Median Δ WJ-MSC Only	Median Δ WJ-MSC + Exosomes	Median Difference	*p*-Value	Effect Size, *r*
ODI	3 months	34	41	−7	−12	−5	0.1018	0.1889
	6 months	22	21	−8	−12	−4	0.0887	0.2596
VAS	3 months	38	42	−4	−4	0	0.8959	0.0146
	6 months	23	21	−4	−3	1	0.5863	0.0821

Table note: Paired *n* refers to the number of patients with both baseline and corresponding follow-up measurements available for each outcome and time point. Δ represents the paired change from baseline, calculated as follow-up value minus baseline value. Negative values indicate improvement, as lower ODI and VAS scores reflect lower functional disability and lower pain intensity, respectively. Median difference was calculated as the median Δ in the WJ-MSC + exosomes group minus the median Δ in the WJ-MSC-only group. Between-group comparisons of paired changes were performed using the Wilcoxon rank-sum test.

**Table 7:** Crude and adjusted relative risks for clinically meaningful improvement according to treatment group.

Outcome	Time Point	Total *n*	WJ-MSC + Exosomes *n*/*N* (%)	WJ-MSC Only *n*/*N* (%)	Crude RR (95% CI)	Crude *p*-Value	Adjusted RR (95% CI)	Adjusted *p*-Value
VAS responder ≥4-point reduction	Any follow-up	86	24/42 (57.14%)	30/44 (68.18%)	0.838 (0.602–1.167)	0.2951	0.836 (0.594–1.175)	0.3021
	3 months	80	24/42 (57.14%)	25/38 (65.79%)	0.869 (0.613–1.230)	0.4275	0.831 (0.576–1.200)	0.3234
	6 months	44	9/21 (42.86%)	12/23 (52.17%)	0.821 (0.437–1.542)	0.5406	0.705 (0.358–1.386)	0.3107
ODI responder ≥10-point reduction	Any follow-up	82	32/42 (76.19%)	20/40 (50.00%)	1.524 (1.071–2.169)	**0.0194**	1.525 (1.075–2.162)	**0.0180**
	3 months	75	27/41 (65.85%)	14/34 (41.18%)	1.599 (1.011–2.529)	**0.0446**	1.462 (0.924–2.313)	0.1048
	6 months	43	17/21 (80.95%)	9/22 (40.91%)	1.979 (1.149–3.407)	**0.0138**	2.003 (1.175–3.414)	**0.0107**

Table note: Values are presented as number of responders/total number of evaluable patients (%). The WJ-MSC + exosomes group was considered the exposed group, and the WJ-MSC-only group was considered the reference group. Crude and adjusted relative risks (RRs) with 95% confidence intervals (CIs) were estimated using Poisson regression models with robust variance. Crude models included treatment group as the only independent variable, whereas adjusted models included treatment group, age, and sex. Bold *p*-values represent statistical significance, *p* < 0.05.

**Table 8:** Frequency of Reported Adverse Events by treatment group and follow-up time point.

Follow-Up Period	Adverse Events in the MSC Only Group (*n* = 133/228, 58.33%)	Adverse Events in the MSC + Exosomes Group (*n* = 95/228, 41.67%)
24–72 h (*n* = 190)	Local: Pain at the puncture site (*n* = 47) Back pain (*n* = 19) Swelling (*n* = 3) Stiffness (*n* = 36) Ecchymosis (*n* = 1)	Local: Pain at the puncture site (*n* = 34) Back pain (*n* = 8) Swelling (*n* = 2) Stiffness (*n* = 31)
	Systemic: Nausea (*n* = 2) Headache (*n* = 2) Fever (*n* = 1) Chills (*n* = 1) Night sweats (*n* = 1)	Systemic: Nausea (*n* = 2)
15 days (*n* = 28)	Local: Back pain (*n* = 6) Pain at the puncture site (*n* = 4) Soreness/sore (*n* = 1) Triggered ball of nerves (*n* = 2)	Local: Back pain (*n* = 3), Pain at the puncture site (*n* = 3) Soreness/sore (*n* = 2)
	Systemic: Limbs pain (*n* = 1)	Systemic: Headache (*n* = 2) Nausea (*n* = 1) Muscle spasms (*n* = 1) Tingling or numbness (*n* = 1) Mucus in stool (*n* = 1)
3 months (*n* = 7)	Local: New and persistent back pain (*n* = 4)	Local: New and persistent back pain (*n* = 2)
		Systemic: Increased neuropathic pain (*n* = 1)
6 months (*n* = 3)	Local: New and persistent back pain (*n* = 1)	Systemic: Increased pain radiation to the left lower extremity (*n* = 1)
	Systemic: Increased pain radiation to the left lower extremity (*n* = 1)	

Table note: Data are presented descriptively as frequencies (*n*) of adverse events, not patients. A total of 228 adverse events were reported, of which 133 (58.3%) occurred in the MSC-only group and 95 (41.7%) in the MSC + Exosomes group. Patients may have experienced more than one adverse event. “Other” refers to adverse events that could not be classified as local or systemic because they were not individually specified in the reporting system.

## Data Availability

The original contributions presented in this study are included in the article/Supplementary Materials. Further inquiries can be directed to the corresponding author.

## Supplementary Materials

The supporting information can be downloaded at: https://www.mdpi.com/article/10.3390/biomedicines14102235/s1.

## Abbreviations

The following abbreviations are used in this manuscript:

AEs	Adverse events
AF	Annulus fibrosus
aRR	Adjusted Risk Ratio
BP	Biological Process
CC	Cellular Component
CEP	Cartilaginous endplate
CI	Confidence Interval
DMEM	Dulbecco’s Modified Eagle Medium
DPBS	Dulbecco’s phosphate-buffered saline
ECM	Extracellular matrix
EVs	Extracellular vesicles
GO	Gene Ontology
hPL	Human platelet lysate
MF	Molecular Function
MMPs	Matrix metalloproteinases
MSCs	Mesenchymal stem cells
NP	Nucleus pulposus
NTA	Nanoparticle tracking analysis
ODI	Oswestry Disability Index
Q1	First Quartile
Q3	Third Quartile
RNA	Ribonucleic acid
ROS	Reactive oxygen species
RR	Risk Ratio
SAE	Serious Adverse Event
SEC	Size exclusion chromatography
STROBE	Strengthening the Reporting of Observational Studies in Epidemiology
TFF	Tangential flow filtration
VAS	Visual Analog Scale
WJ-MSCs	Wharton’s jelly-derived mesenchymal stem cells
