# Supplementary material for: Mechanisms of Exosome-Supplemented WJ-MSC Therapy in Degenerative Disc Disease: Integrating Proteomic Profiling with Clinical Functional Recovery
Source: Biomedicines. 2026 Oct 2;14(10):2235. doi: 10.3390/biomedicines14102235 (PMC13701607; doi:10.3390/biomedicines14102235)
Supplement: Supplementary file 1 [file biomedicines-14-02235-s001.zip › Figure S1.pdf]

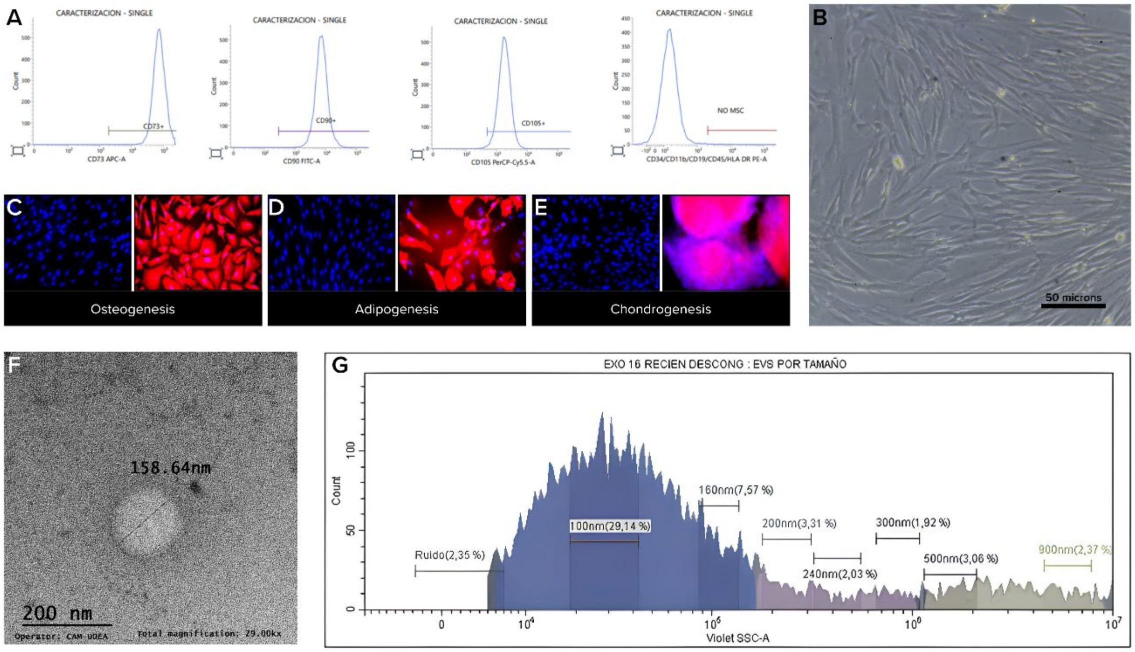

Figure S1. Characterization and quality control of WJ-MSCs and extracellular vesicles.

(A) Flow cytometric characterization of WJ-MSCs showing positive expression of CD73, CD90, and CD105, with minimal expression of the hematopoietic and immune markers CD34, CD19, CD45, CD14, CD11b, and HLA-DR. **(B)** Representative bright-field microscopy image of cultured WJ-MSCs showing their characteristic spindle-shaped morphology. **(C–E)** *In vitro* multilineage differentiation of WJ-MSCs into osteogenic, adipogenic, and chondrogenic lineages, respectively. **(F)** Representative transmission electron microscopy image of an extracellular vesicle with a measured diameter of approximately 158.6 nm. **(G)** Nanoparticle tracking/flow cytometric analysis showing the size distribution of the isolated extracellular vesicle preparation. Together, these results support the identity, multipotency, and physicochemical characterization of the WJ-MSC and EV products used in the study.
